# Myocysticercosis as a Rare Cause of Hand Swelling involving Thenar Group of Muscles: A Case Report

**DOI:** 10.31729/jnma.5101

**Published:** 2020-08-31

**Authors:** KC Saugat, Gaurav Neupane, Anand Regmi, Sujit Shrestha

**Affiliations:** 1 Department of Orthopaedics, Chitwan Medical College Teaching Hospital, Bharatpur, Nepal

**Keywords:** *cysticercosis*, *excision*, *solitary cyst*, *symptomatic*

## Abstract

Larval form of *Taenia solium* causes cysticercosis that commonly involves the central nervous system. Other sites of manifestation are eye, gastrointestinal system, skeletal muscles and subcutaneous tissues. Isolated muscular involvement is rare with only a handful of cases reported in the literature. We present this case of an isolated symtomatic hand swelling due to Myocysticercosis which pose diagnostic dilemma. This should be considered in differential diagnosis in our developing nation and especially in endemic region. High resolution ultrasound of the hand (thenar region) helped in the diagnosis and is often diagnostic like in our case. The treatment of choice of an isolated symptomatic lesion without involvement of central nervous system is surgical excision which we did followed by short course of antiheminthic and anti-inflammatory medication for two weeks.

## INTRODUCTION

Cysticercus cellulosae is the larval form of Taenia solium which causes cysticercosis.^1^ It is endemic in certain developing countries of Africa, Eastern Europe, Mexico, and South-East Asian regions.^2^ Central nervous system is commonly involved in this disease, but it may affect eyes, subcutaneous tissues, liver, gastro-intestinal system, skeletal muscle, and at times lung and heart.^3^ Symptomatic isolated lesion in thenar muscle is very rare entity reported in very few literatures. High resolution ultra sound is a reliable diagnostic measure for the isolated intramuscular and subcutaneous lesion. It is safe, non-ionising, cost-effective and widely-available imaging tool for diagnosis of Myocysticercosis.

## CASE REPORT

A 36 years old female from Gandaki Province (Nawalparasi), Nepal presented to our Orthopaedic outpatient department with progressive swelling over the palmar aspect of left hand over thenar eminence for 2 months duration. Intially it was pain free but since two weeks she developed pain which was progressive mild to moderate in intensity and dull aching nature and continous type. There was no history of fever, trauma, seizure and fits, sensory or motor deficit in affected hand.

**Figure 1.**
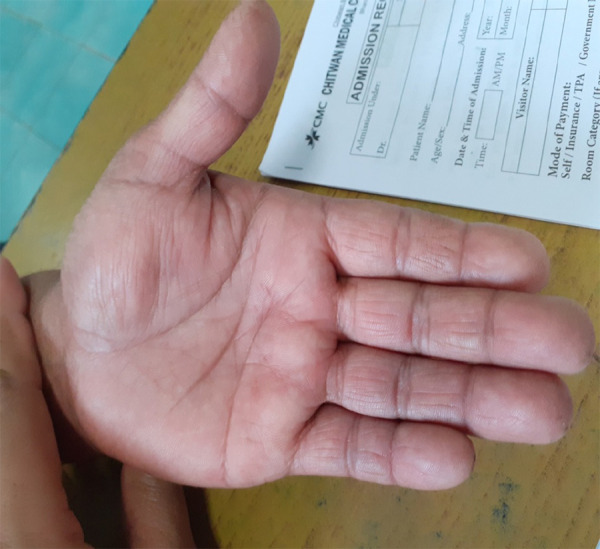
Swelling over left thenar of size 2x3 cm.

On examination there was a 2×3 cm swelling over the thenar eminence of her left hand with normal overlying skin. Swelling was soft, mild tenderness with normal surface temperature. The surface was smooth, margins were ill defined and fluid thrill was absent. The swelling was fixed to underlying structures but overlying skin was free (Figure 1).

**Figure 2.**
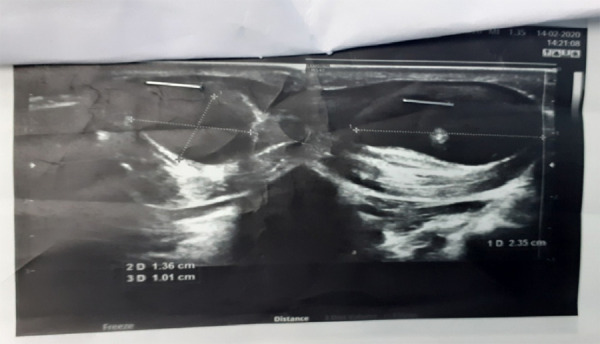
Ultrasound findings showing defined cyst measuring 1.5×2.5×1.0 cm within thenar muscle with intracystic echogenic contents and tiny cysts.

Sonography of left hand was suggestive of a well defined cyst measuring 1.5×2.5×1.0 cm within thenar muscle with intracystic echogenic contents and tiny cysts within with adjacent inflammatory changes suggesting feature of Myocysticercosis. No vascularity noted on colour Doppler. On changing position of left hand this focus (nidus) showed mobility. (Figure 2)

**Figure 3.**
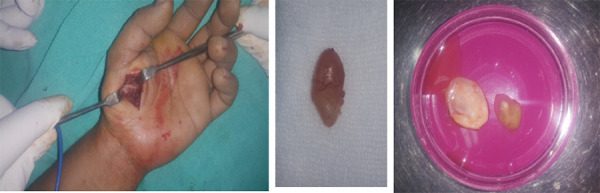
A,B,C. Intraoperative picture of excision, Icyst in total and its content inside.

On the basis of above findings diagnosis of Myocysticercosis was made. There were no neurological features and ophthalmologic examination (vision and indirect opthalmoscopy) was normal. It was an isolated symptomatic lesion so we excised the lesion in total (Figure 3).

**Figure 4.**
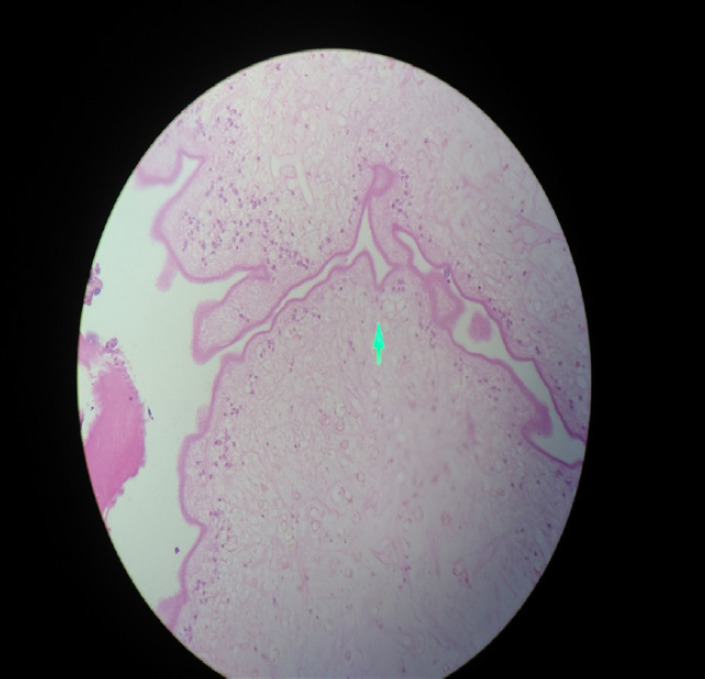
Histopathological findings of Myocysticercosis.

This was also later confirmed by histopathological evaluation as shown (Figure 4). Due consent has been taken from the patient. Patient was discharged with antihelminthic medication (Albendazole for two weeks), analgesic and steroid. She presented to our OPD for suture removal after one week and was uneventful.

## DISCUSSION

Cysticercosis is a parasitic infection transmitted to the human beings by ingestion of eggs from contaminated food or water. 86% of the cases are seen in brain and eyes while the remaining 14% occur in subcutaneous, pulmonary, cardiac, muscular, hepatic and oral locations. Humans are the only definitive host for Taenia solium whereas pigs are usual intermediate host along with dogs, cats and sheep.^4^ Many factors like geographical location, ethnic group, religion, socioeconomic status, education, food habits, hygiene and sanitation and standard of living are associated with the disease burden.^5^ In the context of Nepal, the majority of population belonging to Rai, Limbu, Magar and Sarkies ethnic groups, taenia infestation is high. It is due to rearing of pigs, high consumption of pork, poor sanitary measures and poor socio-economic conditions.^6^ Taenia solium, is becoming an increasing problem in Nepal with high prevalences of porcine cysticercosis and human taeniasis/cysticercosis detected in epidemiological studies undertaken in different parts of the country. Pig farming and marketing have increased dramatically in the country in recent years due to increased consumer demand for pork as the country's caste system has become relaxed. Postmortem surveys of pigs at slaughter establishments in Kathmandu and Dharan municipality showed 14% (34/250

Myocysticercosis have been described as three different types in the literature. The first type is due to leakage of cyst fluid causing inflammatory pain. This is known as Myalgic type. Myopathic type is chronic degenerative type of cyst which is caused by minimal leakage with chronic inflammatory mass or abscess like swelling. The third type is Pseudo-hypertrophy type in which multilocular cyst formation occurs in a group of muscles.^5^ Our case was Myalgic type.

The different modality of investigation are Ultrasound, Computed Tomography (CT) and Magnetic Resonance Imaging (MRI). Diagnosis of the lesion in our case was made by High resolution Ultrasound. It is a safe, non-ionising, non-invasive, cost-effective and widely available modality of investigation by which definitive diagnosis can be ascertained confidently. The salient diagnostic feature is that of the cysticercus itself, which appears as an oval or round well-defined cystic lesion with an eccentric echogenic scolex in it.^7^

Taenia solium infestation is a preventable disease in the community. Prevention of community outbreak can be done by good personal hygiene, proper hand washing and sanitation. Raw vegetables and salads should be washed properly with safe water before consumption and proper fecal disposal is essential. Deworming in regular period and adequate cooking of pork meat is requirement for killing intestinal worms and cysticercosis larva. Treatment depends on numerous factors like the site, number of cysts and presence of symptoms. Isolated myocysticercosis usually requires no treatment unless it is painful which requires surgical excision.^8^
